# Flexural band gaps and response attenuation of periodic piping systems enhanced with localized and distributed resonators

**DOI:** 10.1038/s41598-019-56724-0

**Published:** 2020-01-09

**Authors:** Mohd Iqbal, Mahesh Murugan Jaya, Oreste Salvatore Bursi, Anil Kumar, Rosario Ceravolo

**Affiliations:** 1 Department of Mechanical and Industrial Engineering, Indian Institute of Technology Roorkee, Roorkee, 247667 India; 2 Department of Civil, Environmental and Mechanical Engineering, University of Trento, Trento, 38123 Italy; 3 Department of Structural and Geotechnical Engineering, Politecnico di Torino, Torino, 10129 Italy

**Keywords:** Civil engineering, Mechanical engineering

## Abstract

Novel metamaterial concepts can be used to economically reduce flexural vibrations in coupled pipe-rack systems. Here, we model pipe on flexible supports as periodic systems and formulate dispersion relations using Floquet-Bloch theory which is verified by a finite element model. Owing to the flexibility of the coupled system, a narrow pass band is created in low frequency regime, in contrast to the case of pipe without any rack. Two types of vibration reduction mechanisms are investigated for pipe with different supports, i.e. simple and elastic support. In order to tune the band gap behaviour, lateral localized resonators are attached at the centre of each unit cell; conversely, the lateral distributed resonators are realized with a secondary pipe existing in the system. The results reveal that both Bragg and resonance type band gaps coexist in piping systems due to the presence of spatial periodicity and local resonance. Although, the response attenuation of a coupled pipe-rack system with distributed resonators is found to be little lower than the case with the localized one, the relatively low stiffness and damping values lead to cheaper solutions. Therefore, the proposed concept of distributed resonators represents a promising application in piping, power and process industries.

## Introduction

**Figure 1:**
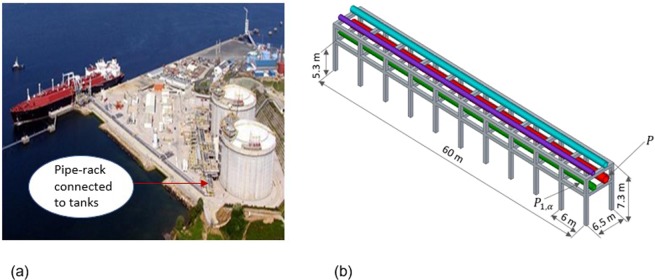
Layout of a typical LNG plant. (**a**) A pipe-rack connected to tanks. (**b**) Schematic of a typical periodic rack containing pipes of different dimensions, where $P$ and ${P}_{1,\alpha }$ denote main and secondary pipes, respectively.

Pipes conveying fluid supported in equally spaced racks are very common in liquefied natural gas (LNG) plants, thermal power plants, petroleum industries, chemical plants and in many other engineering applications. LNG plant consists of many units such as gas receiving terminals, pipelines, storage tanks, etc. Long pipelines in such plants are used to carry refrigerated liquefied gas to storage tanks and shipping terminals. Excessive vibrations of pipelines due to ambient load, flow pulsation, valve or support excitation can result in fatigue damage, loosening of connections, etc., which may lead to fire, explosion, safety and environmental issues. It is thus essential to protect them from large vibration amplitude. To crystallize the idea, an LNG plant containing a coupled pipe-rack system connected to a tank^1^ is shown in Fig. 1a. Such a system usually contains pipes of different dimensions supported on a finite periodic rack as highlighted in Fig. 1b.

Periodic structures have been used as a common tool for mitigation of acoustic and elastic waves over the past decades^2–5^. Periodicity in a structure may be in one, two or in all the three dimensions^6^. Such systems exhibit unique frequency band gap characteristics^3^, which can be generated either due to the Bragg scattering^6,7^ or by local resonances^8^. As a result, they allow only waves of a certain frequencies to pass through, which are represented as pass or propagation bands. The remaining frequencies get attenuated, thereby forming stop or non-propagation bands. If the spatial periodicity of a structure is comparable to the wavelength λ, then Bragg band gaps are induced in the structure and appear around the frequencies governed by the Bragg condition $l=n(\frac{\lambda }{2}),{\rm{where}}\,n=1,2,3,\ldots$ and *l* is length of the unit cell. Studies were previously conducted on band gaps in finite^9^, semi-infinite^5,10^ and infinite^3,4,11^ periodic structures caused by Bragg scattering. Moreover, analytical, numerical and experimental investigations on Bragg band gaps in periodic structures have been carried out by several researchers^12–16^.

In recent studies, metamaterial beams, shafts and rods endowed with periodic resonators have been investigated. In order to filter undesired longitudinal^17^, flexural^18–26^ or torsional^27,28^ waves, a periodic structure can be equipped with resonator units that entail new band gaps different from those produced by Bragg scattering^7^. The introduction of damping devices in such resonator units contributes to energy dissipation, thereby reducing vibration amplitude^29^. In order to achieve an efficient energy dissipation, the lateral localized resonators (LLRs) or tuned mass dampers (TMDs) have to be optimally designed^30,31^. This is achieved by adjusting their frequency and damping ratios so as to minimize some significant response quantity of the main system, e.g. displacement. In the context of vibration mitigation in pipes, tuned mass dampers with different energy dissipation mechanisms such as material damping in the damper element of TMD^32,33^, fluid damping in TMD mass^34^ or impact of TMD mass with a dissipative surface^35^ were developed. All adopted damper technologies require the use of an external mass, indeed, which needs to be connected to a pipe using a spring-damper system. Clearly, this solution results to be less practical.

In a typical pipe-rack system, it is common to have pipes of different dimensions. It may thus be possible to conceive a pipe of smaller cross section, see *P*_1,α_ in Fig. 1b, as a lateral distributed resonator (LDR) system. The dynamic characteristics of *P*_1,α_ and its effects on a coupled pipe-rack system are different from those of LLRs and are the topics explored in the paper further in conjunction with the periodicity effects.

Along these lines, the propagation of flexural waves is studied in a pipe-rack system similar to the case study shown in Fig. 1. Two models are considered in this regard: (i) a pipe without rack, i.e. a pipe on simple supports -Type #1-; and (ii) a pipe with a rack, i.e. a pipe on elastic supports -Type #2-. Type #2 support approximates a realistic scenario while Type #1 represents an extreme case. As a result, flexural wave propagation in an undamped long elastic pipe *P* on Type #1 and Type #2 supports is examined, both analytically and numerically. Thus, in order to determine analytical dispersion relations, the Floquet-Bloch theory of periodic systems is employed.

Nonetheless, it is not straightforward to derive similar dispersion relations when material damping in a pipe *P* is taken into account or when either a LLR or a LDR (*P*_1,α_) is attached to *P*. Hence, a finite element (FE) model is set for these studies, the accuracy of which is verified by comparing an undamped finite element model of *P* with analytical results. Optimal stiffness and damping values are then designed for the case when *P* is connected to a LLR and to LDR or *P*_1,α_. Moreover, the effect of damping in resonators on band gap characteristics is investigated by means of the transfer function of *P* provided by the FE model of the coupled system. Finally, the vibration attenuation of *P* by means of LLRs is compared with the case when LDRs with the same mass ratio α, i.e. *P*_1,α_ is used. Though the performance of a coupled pipe-rack system with LDRs is less efficient than the case with LLRs, relevant low stiffness and damping values lead to cost-saving solutions.

## Results

### Wave propagation in an undamped pipe

**Figure 2:**
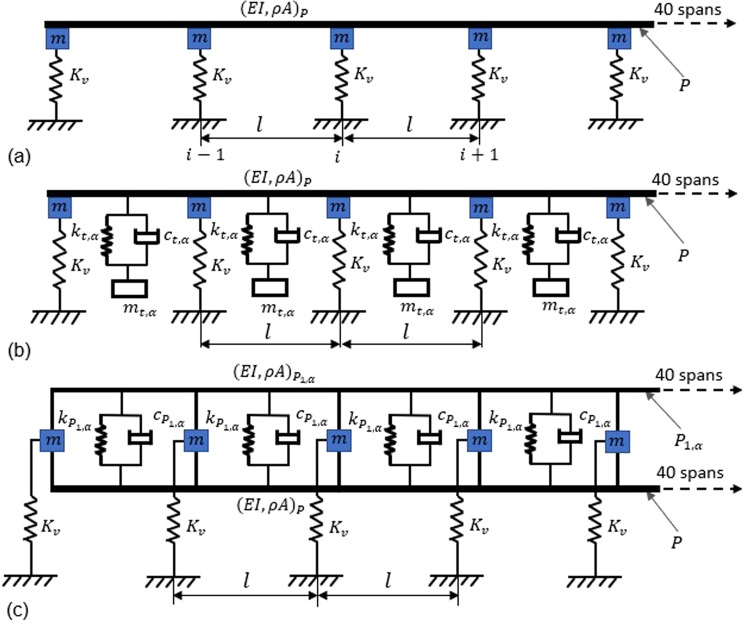
Models for a pipe $P$ of Fig. 1b on a periodic rack, i.e. a pipe on elastic supports- Type #2-. (**a**) Uncontrolled pipe *P*. (**b**) Pipe $P$ with lateral localized resonator (LLR) at the centre of each unit cell. (**c**) Pipe $P$ connected to lateral distributed resonator (LDR), i.e. pipe ${P}_{1,\alpha }$.

The dispersion relation for an undamped pipe *P* of unit cell length *l*, cross sectional area *A*, moment of inertia *I*, density *ρ* and Young’s modulus *E* with Type #2 support condition shown in Fig. 2a is derived based on the Floquet-Bloch theorem^36,37^. The analytical expression of dispersion relation between the circular frequency ω and the propagation constant *μ* (i.e., i*kl*) is given by

1 $$\psi {\cosh }^{2}\mu +\chi \cosh \,\mu +\eta =0$$

where

$$\psi =[\{\cosh (\varOmega l)-\,\cos (\varOmega l)\}{]}^{2}-\{{\sinh }^{2}(\varOmega l)-{\sin }^{2}(\varOmega l)\}]$$

$$\chi =\left[\begin{array}{c}\{\,\sinh (\varOmega l)-\,\sin (\varOmega l)\}\{\,\cosh (\varOmega l)\,\sin (\varOmega l)+\,\cos (\varOmega l)\sinh (\varOmega l)\}\\ -\{\,\cosh (\varOmega l)\,\sin (\varOmega l)-\,\cos (\varOmega l)\sinh (\varOmega l)\}\{\,\sin (\varOmega l)+\,\sinh (\varOmega l)\}\\ +\frac{12\{\sinh (\varOmega l)-\,\sin (\varOmega l)\,\{1-\,\cos (\varOmega l)\cosh (\varOmega l)\}\{{K}_{v}-m{\omega }^{2}\}}{{(\varOmega l)}^{3}}\end{array}\right]$$

$$\eta =\left[\begin{array}{c}\{{\sin }^{2}(\varOmega l){\cosh }^{2}(\varOmega l)-{\cos }^{2}(\varOmega l){\sinh }^{2}(\varOmega l)\}-{\{\cosh (\varOmega l)-\cos (\varOmega l)\}}^{2}\\ +\frac{12\{\cosh (\varOmega l)\sin (\varOmega l)-\,\cos (\varOmega l)\sinh (\varOmega l)\}\{1-\,\cos (\varOmega l)\cosh (\varOmega l)\}\{{K}_{v}-m{\omega }^{2}\}}{{(\varOmega l)}^{3}}\end{array}\right]$$

and

$$\varOmega ={\left(\frac{\rho A{\omega }^{2}}{EI}\right)}^{\frac{1}{4}}$$

See the section ‘Methods’ for detailed derivation. The analytical dispersion relation given in Eq. (1) is obtained by solving Eq. (19).

The behaviour of the pipe-rack coupled system can be characterised by these dispersion relations. Equation (1) is quadratic in cosh*μ* and thus yields two pairs of distinct roots of *μ*; ±*μ*_1_ and ±*μ*_2_ for each frequency *ω*. Positive and negative signs of *μ* describe the same characteristics of wave motion travelling in opposite directions. Generally, *μ* is complex and can be written as

2 $$\mu =\delta +{\rm{i}}\gamma $$

where, the real part 𝛿 describes rate of attenuation of amplitude and imaginary part *γ* imparts information about the phase difference of travelling wave between two adjacent unit cells. If the Bloch wave number of a freely propagating non-decaying wave (*δ* = 0) is *k*, then

3 $$k=\frac{\gamma }{l}$$

and is related to the corresponding wavelength *λ* as

4 $$\lambda =\frac{2\pi }{k}=\frac{2\pi l}{\gamma }$$

Based on the nature of *μ*, there are three types of wave. For $\delta  > 0$, there will be a decay in the amplitude and hence no energy flows in the direction of wave propagation, and are defined as attenuating/evanescent waves. In this case, the adjacent unit cells vibrate either in phase or out of phase. On the other hand, if $\mu$ is purely imaginary, energy flows in the direction of propagation and the waves pass without any attenuation thereby exhibiting only pass band in the dispersion curves. For a complex $\mu$, a part of the energy propagates while the remaining gets attenuated, which results in the occurrence of both pass and stop bands in the dispersion curves.

When ${K}_{v}\to \infty$ and $m=0$, the analytical expression of dispersion relation given in Eq. (1) leads to the case of $P$ on Type #1 support. The resulting expression of dispersion relation is given by,

5 $$\cos (kl)=\,\cosh (\mu )=\frac{\cosh (\varOmega l)\sin (\varOmega l)-\,\cos (\varOmega l)\sinh (\varOmega l)}{\sin (\varOmega l)-\,\sinh (\varOmega l)}$$

which is similar to those found in literature^3^.

For the analytical flexural wave propagation study, a pipe $P$ with $l=6\,m$, outer diameter of $406.40\,mm$ and thickness $7.92\,mm$ is used. Young’s modulus and density are assumed to be 200 *GPa* and 7800 *kg*/m^3^, respectively.

**Figure 3:**
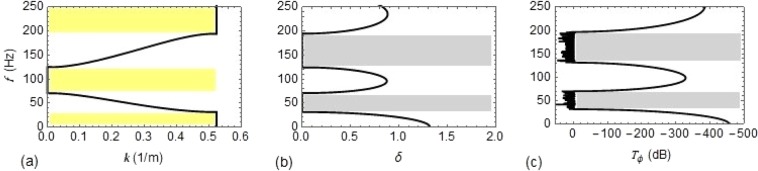
Dispersion curves and frequency response for Type #1 support: (**a**) as a function of the Bloch wave number $k$; (**b**) as a function of the real part of $\mu$; (**c**) as a function of ${T}_{\phi }\,(dB)$.

Equation (5) is used to obtain the variation of $k$ and $\mu$ with frequency of the wave *f* = 2π/*ω*, and is shown in Fig. 3a,b, respectively. Frequency ranges of first two stop bands are $[0-31.36]\,Hz$ and $[69.63-131.2]\,Hz$ and are represented by yellow shaded region in Fig. 3a. The pass bands, between these frequencies, are represented by grey shaded region in Fig. 3b,c.

Now, wave propagation characteristics in pipe *P* with Type #2 support as shown in Fig. 2a is examined. For this case, a typical concrete rack structure of C50/40 class is considered. Each frame of the rack is 6.5 m wide and 7.3 m tall with 6 m spacing between adjacent frames along the length of *P*. Each frame consists of two storeys, placed at a level of 7.3 m and 5.3 m from the ground. Each column of the rack is of 600 mm^2^. The pipe-rack structure consists of 40 frames of which the first 10 are shown in Fig. 1b. A simplified numerical model using spring *K*_*v*_ and lumped mass *m*, as shown in Fig. 2a, is made such that its first mode matches with the first lateral mode of the pipe-rack structure obtained using a complete FE model. It is observed that the first mode occurs at 4.45 Hz and based on the corresponding mass participation, *m* and *K*_*v*_ are calculated to be 22.88 *T* and 17.9 MN/m, respectively.

**Figure 4:**
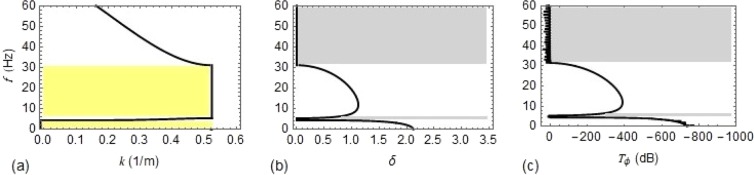
Dispersion curves and frequency response for Type #2 support: (**a**) as a function of Bloch wave number $k$; (**b**) as a function of the real part of $\mu$; (**c**) as a function of ${T}_{\phi }\,(dB)$.

Based on the dispersion relation from Eq. (1), the variation of *k* and *μ* with frequency of the wave *f* is obtained and is shown in Fig. 4a,b, respectively. In contrast to Type #1 support, a narrow pass band is generated near 4.45 Hz, which is the first predominant natural frequency of the rack structure. In the frequency range from $0$ to 60 *Hz*, there are two stop bands with the range [0−4.38] *Hz* and [5.34−31.5] *Hz*, represented by yellow shaded region in Fig. 4a. Similarly, the pass bands are represented by grey shaded region in Fig. 4b,c.

To verify the analytical results, FE modelling of *P* on Type #1 and Type #2 supports consisting of 40 unit cells is used. A harmonic excitation in the form of rotation of unit amplitude is applied at the left end of *P* and the steady state frequency response is extracted at the right end. *T*_*ϕ*_ is calculated from Eq. (20), which is plotted in Figs. 3c and 4c for Type #1 and Type #2 supports respectively, showing excellent agreement with the analytical results. The real part of *μ* (Figs. 3b and 4b) and *T*_*ϕ*_ (Figs. 3c and 4c) reports about the attenuation behaviour, while the Bloch wave number (Figs. 3a and 4a) refers to the propagation behaviour.

### Vibration in a controlled periodic pipe

An identical single mass LLR of mass ${m}_{t,\alpha }$, stiffness ${k}_{t,\alpha }$ and damping coefficient ${c}_{t,\alpha }$ is attached as a substructure at midpoint ($l/2$) of each unit cell of $P$ for both Type #1 and Type #2 supports. Similarly, ${P}_{1,\alpha }$ of mass ratio $\alpha$ which is present in the rack is attached to $P$ using spring-damper system with stiffness ${k}_{{P}_{1,\alpha }}$ and damping coefficient ${c}_{{P}_{1,\alpha }}$ at the centre of each unit cell. Figure 2b shows the configuration of $P$ with LLRs for Type #2 support while Fig. 2c shows the case when ${P}_{1,\alpha }$ is attached as LDR. Material damping ratio $\xi$ for both the pipes $P$ and ${P}_{1,\alpha }$ is assumed to be 0.02. In order to assess the performance, three mass ratios of 0.05, 0.16 and 0.25 are chosen and the corresponding dimensions of ${P}_{1,\alpha }$ are detailed in Table 1.

**Table 1:** Dimensions of *P*_1,α._

Pipe	Outer diameter (*mm*)	Thickness (*mm*)	Mass per unit length (*kg*/*m*)
${P}_{1,\alpha =0.05}$	60.33	2.77	3.93
${P}_{1,\alpha =0.16}$	152.40	3.40	12.44
${P}_{1,\alpha =0.25}$	219.80	21.56	79.94

Optimization is performed to reduce the vibration response till $60\,Hz$ using Eq. (21), based on which the optimal ${k}_{t,\alpha }$ and ${c}_{t,\alpha }$ for Type #1 and Type #2 supports are calculated. Equation (22) is used to calculate the corresponding performance index $Z$ for all the cases, a smaller value of which denotes better performance in terms of vibration reduction. Similarly, the optimal ${k}_{{P}_{1,\alpha }}$ and ${c}_{{P}_{1,\alpha }}$ are evaluated for the case when ${P}_{1,\alpha }$ is connected to $P$. Table 2 contains the optimal values for both scenarios. The equivalent frequency ${f}_{t,\alpha }$ of the LLR and the corresponding damping ratio ${\xi }_{t,\alpha }$ are provided in Table 3. Since ${P}_{1,\alpha }$ is a continuous system with multiple modes, its frequency and damping ratios are not reported.

**Table 2:** Optimal stiffness and damping values.

Support condition		Type #1			Type #2		
*α*		0.05	0.16	0.25	0.05	0.16	0.25
*P* with LLR	${k}_{t,\alpha }\,(N/m)$	1.30E6	2.87E6	3.05E6	1.37E6	3.05E6	3.99E6
	${c}_{t,\alpha }\,(Ns/m)$	3.59E3	1.19E4	2.44E4	3.16E3	1.09E4	1.80E4
	$Z$	2.08E-3	8.03E-7	6.77E-8	2.83E-3	1.10E-6	4.94E-9
*P* with ${P}_{1,\alpha }$	${k}_{{P}_{1,\alpha }}\,(N/m)$	1.05E1	2.46E6	1.47E6	1.53E1	2.49E6	1.72E6
	${c}_{{P}_{1,\alpha }}(Ns/m)$	1.51E3	6.38E3	8.17E3	9.51E2	6.74E3	7.07E3
	$Z$	8.93E-1	7.61E-3	2.55E-4	8.97E-1	8.93E-3	2.55E-4

**Table 3:** Optimal frequency and damping ratio for LLR.

Support condition	Type #1	Type #2				
$\alpha$	$0.05$	$0.16$	$0.25$	$0.05$	$0.16$	$0.25$
$\,{f}_{t,\alpha }\,(Hz)$	37.73	31.32	25.58	38.68	32.25	29.27
ξ_*t,α*_	0.33	0.41	0.64	0.28	0.36	0.41

**Figure 5:**
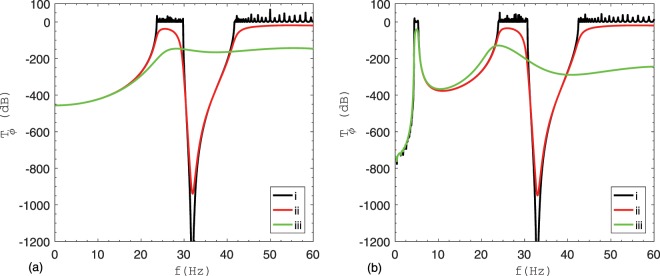
${T}_{\phi }$ of $P$ controlled with LLRs for various damping values: (**a**) Type #1 support; (**b**) Type #2 support.

The frequency response of only the case with $\alpha =0.16$ is reported here as others show similar behaviour. In order to understand the effect of damping on band gaps when a LLR is used, three cases are considered for both types of supports; (i) material damping in $P$ $(\xi =0)$ and the damping coefficient of LLR ${c}_{t,\alpha }$ are neglected (${k}_{t,\alpha }=2.87E6$, ${c}_{t,\alpha }=0$ for Type #1 and ${k}_{t,\alpha }=3.05E6$, ${c}_{t,\alpha }=0$ for Type #2), (ii) material damping in $P$ $(\xi =0.02)$ is considered while the damping coefficient of LLR ${c}_{t,\alpha }$ is neglected (${k}_{t,\alpha }=2.87E6$, ${c}_{t,\alpha }=0$ for Type #1 and ${k}_{t,\alpha }=3.05E6$, ${c}_{t,\alpha }=0$ for Type #2) and (iii) both material damping in $P$ $(\xi =0.02)$ and the damping coefficient of LLR ${c}_{t,\alpha }$ are considered (${k}_{t,\alpha }=2.87E6$, ${c}_{t,\alpha }=1.19E4$ for Type #1 and ${k}_{t,\alpha }=3.05E6$, ${c}_{t,\alpha }=1.09E4$ for Type #2). Figure 5a shows the response for above three cases with Type #1 support while Fig. 5b shows the same for Type #2 support.

When both $P$ and LLR are undamped, a new band gap at the natural frequency of LLR is created and thus two types of band gap coexist. In case of Type #1 support, $[0-23.64]\,Hz$ and $[29.82-41.64]\,Hz$ represents the Bragg and resonance stop band, respectively. For Type #2 support, $[0-4.38]\,Hz$ and $[5.34-23.88]\,Hz$ are the Bragg stop bands while $[30.72-42.42]\,Hz\,$is the resonance stop band.

**Figure 6:**
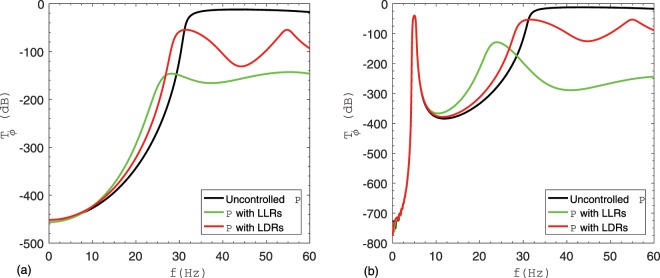
Comparison between ${T}_{\phi }$ of $P$ when coupled to LLRs and to LDRs (${P}_{1,\alpha }$) respectively, for $\alpha \,=\,0.16$: (**a**) Type #1 support; (**b**) Type #2 support.

The reduction in amplitude of vibration of $P$ is compared when ${P}_{1,\alpha =0.16}$ is used as LDRs instead of the LLR of $\,\alpha =0.16$. For this, the corresponding frequency response plots are shown in Fig. 6a,b, for Type #1 and Type #2 supports, respectively. As expected, significant reduction in vibration is achieved for the case of LLR, while ${P}_{1,\alpha }$ is found to be less efficient for the same mass ratio. More precisely, with LLRs/LDRs attached to the pipe P, the total mass of the system is increased and natural frequencies of the system are decreased. As a consequence, the width of a stop band is reduced and this reduction is higher in case of LLRs as compared to the LDRs. In fact, in the case of LLRs the mass is concentrated in the middle of each pipe span and the relevant participating mass is higher than the case with a distributed mass. Furthermore, both LLRs and LDRs are introduced to reduce the vibration amplitude of propagation waves in the first pass band for Type #1 support and in the second pass band for Type #2 support, respectively. This is achieved by means of the optimal stiffness and damping values reported in Table 2 for $\alpha =0.16$. Owing to dynamic effects of LLRs and LDRs, one can note the shrinking of stop bands and reduction of amplitudes in pass bands in Fig. 6.

## Discussion

In order to reduce flexural vibrations in coupled pipe-rack systems of LNG plants, novel and economic ways based on metamaterial concepts are proposed. As a result, flexural wave propagation in periodic piping system is analysed by solving relevant fourth-order equation of motion. Subsequently, the Floquet-Bloch theorem is applied to obtain the analytical dispersion relation, which was used to validate the results provided by a numerical model. The propagation characteristics of pipe $P$ for both types of support conditions, simply supported – Type #1- and with elastic supports –Type #2- are identical except that in case of Type #2 supports, where a new narrow pass band occurs near the first natural frequency of the rack. The lower and upper bounding frequencies of pass bands coincide with the natural frequencies of the coupled system.

Pipes without lateral localized resonators (LLRs) at the centre of each unit cell exhibit only Bragg band gaps. Conversely, the adoption of LLRs without damping entails a new band gap located near the natural frequency of LLRs which can be observed in both Fig. 5a,b for Type #1 and Type #2 support, respectively. The results reveal that both Bragg and resonance type band gaps coexist in piping systems due to the presence of spatial periodicity and local resonance. In addition, the introduction of LLRs increases the whole mass of the system, which results in decreasing the main natural frequencies of the coupled pipe-rack system. More precisely, in the case of Type #2 supports the first Bragg stop band remains the same, while the second stop band shifts to the low frequency range as depicted in Fig. 5b. The presence of damping in LLRs smoothens and lowers the transmission ${T}_{\phi }$ of $P$ and widens the band gap. High damping in LLRs causes the band gap to vanish^21,22^, but can significantly reduce vibration amplitude.

Further, a new way of vibration suppression was investigated by the attachment of a smaller cross section pipe, the so called lateral distributed resonators (LDRs), in parallel to the main pipe $P$. It was observed that, with an increase of the mass ratio $\alpha$ between resonators and pipe, both LLRs and LDRs (${P}_{1,\alpha }$) performed better. This is evident from Table 2, which shows a decrease of the performance index $Z$, defined in Eq. (22), with an increase of $\alpha$. Also, the optimal values of ${k}_{t,\alpha }$ and ${c}_{t,\alpha }$ for LLRs increase with an increase of $\alpha$. For the same $\alpha$, it was found that $Z$ corresponding to the case LDRs was greater than that of the case with LLRs; this can be inferred from both Table 2 and Fig. 6. In fact, for any value of $\alpha$, the optimal ${k}_{{P}_{1,\alpha }}$ and ${c}_{{P}_{1,\alpha }}$ for ${P}_{1,\alpha }$ is less than that for the LLRs case. This is because ${P}_{1,\alpha }$ is a continuous system and, therefore, the whole mass cannot be mobilized for any frequency: this leads to a lower effective mass ratio. Conversely, for the corresponding LLRs case, the complete mass contributes to the frequency for which it is designed^38^. Clearly, the trend of ${T}_{\phi }$ shown in Fig. 6 for ${P}_{1,\alpha =0.16}$ cannot be utilized for other pipes endowed with $\alpha =0.16$. This is because the dynamic characteristics of ${P}_{1,\alpha =0.16}$ depends on radius/thickness ratio. Thus, to determine optimal parameters for other pipes, a rigorous optimization has to be performed again.

In sum, even though the performance of a coupled pipe-rack system with LDRs is less efficient than the case with LLRs, the relevant low stiffness and damping values lead to cheaper solutions. As a result, the adoption of pipes ${P}_{1,\alpha }$ represents a promising application. Eventually, the enhancement of these results by means of nonlinear devices/mechanisms deserves further studies.

## Methods

### Derivation of dispersion relations

**Figure 7:**
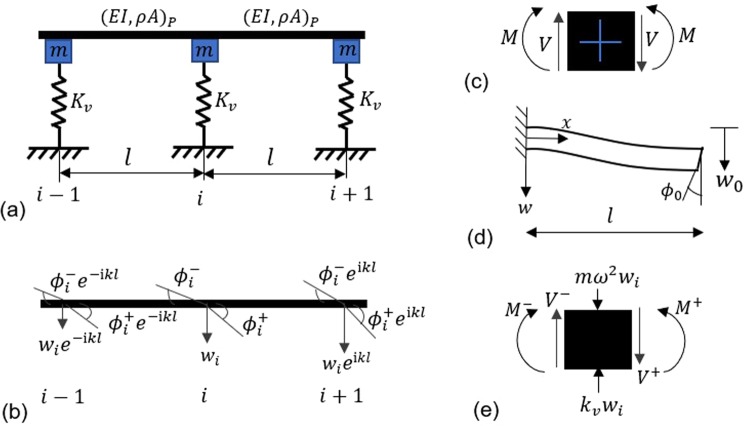
Modelling of pipe for dispersion analysis. (**a**) Pipe on Type #2 support (**b**) Floquet-Bloch theorem of periodic structure applied at the nodes for transverse and angular displacements. (**c**) Illustration of sign conventions for shear forces and bending moments. (**d**) Representation of a single span pipe as a simple beam clamped at one end and displacement ${w}_{0}$ and rotation ${\phi }_{0}$ at free end (**e**) Bending moments and forces balance at the generic node $i.$

The system shown in Fig. 7a is considered for flexural wave analysis which consists of two unit cells each of length $l$. The undamped pipe $P$ is assumed to be an Euler- Bernoulli beam, the equation of which is given as,

6 $$\frac{{\partial }^{2}}{\partial {x}^{2}}\left[EI\frac{{\partial }^{2}w(x,t)}{\partial {x}^{2}}\right]+\rho A\frac{{\partial }^{2}w(x,t)}{\partial {t}^{2}}=0$$

where $\rho$ and $E$ are the density and modulus of elasticity of the material, respectively. $A$ is area of cross-section and *I* is the second moment of inertia of the beam. $w(x,t)$ is the transverse displacement, and $x$ represents the spatial coordinate along the length of beam. The substitution of steady state solution $w(x,t)=w(x){e}^{{\rm{i}}\omega {\rm{t}}}$ in Eq. (6) leads to,

7 $$EI{w}^{IV}(x)-\rho A{\omega }^{2}w(x)=0$$

where $\omega$ is the circular frequency. The solution of Eq. (7) can be written as,

8 $$w(x)={A}_{1}\,\cos (\varOmega x)+{A}_{2}\,\sin (\varOmega x)+{A}_{3}\,\cosh (\varOmega x)+{A}_{4}\,\sinh (\varOmega x)$$

with $$\varOmega ={(\frac{\rho A{\omega }^{2}}{EI})}^{\frac{1}{4}}$$.

Applying Floquet-Bloch theory on each unit cell as shown in Fig. 7b, the transverse displacements of nodes $i+1$ and $i-1$ are related to those at node $i$ as

9 $${{w}}_{i+1}={{w}}_{i}{e}^{{\rm{i}}kl},\,{{w}}_{i-1}={{w}}_{i}{e}^{-{\rm{i}}kl}$$

where, $k$ represents one dimensional Bloch wave number, *l* is the distance between supports and i is $\sqrt{-1}$. The term i*kl* in Eq. (9) is called propagation constant $\mu$. The similar relations are used for rotations.

The constants ${A}_{1}$, ${A}_{2}$, ${A}_{3}$ and ${A}_{4}$ in Eq. (8) are computed by applying boundary conditions shown in Fig. 7d, and are then used to determine the shear forces $V$ and bending moments $M$ on either side of node $i$. Figure 7c shows the sign convections for shear forces and bending moments. The expression for dynamic compliance coefficients^39^ at $x=0$ and $x=l$ for ${w}_{0}=1$ and ${\phi }_{0}=0$ are,

10 $$\begin{array}{c}{V}_{0}^{^{\prime} }=\frac{{\Omega }^{3}EI[\sin (\varOmega l)+\,\sinh (\varOmega l)]}{1-\,\cos (\varOmega l)\cosh (\varOmega l)}\\ {V}_{l}^{^{\prime} }=\frac{{\varOmega }^{3}EI[\cosh (\varOmega l)\,\sin (\varOmega l)+\,\cos (\varOmega l)\sinh (\varOmega l)]}{1-\,\cos (\varOmega l)\cosh (\varOmega l)}\\ {M}_{0}^{^{\prime} }=\frac{{\varOmega }^{2}EI[\cos (\varOmega l)-\,\cosh (\varOmega l)]}{1-\,\cos (\varOmega l)\cosh (\varOmega l)}\\ {M}_{l}^{^{\prime} }=\frac{{\varOmega }^{2}EI[\sinh (\varOmega l)\sin (\varOmega l)]}{1-\,\cos (\varOmega l)\cosh (\varOmega l)}\end{array}$$

and for ${w}_{0}=0$ and ${\phi }_{0}=1$ are given by

11 $$\begin{array}{ccc}{V}_{0}^{^{\prime\prime} } & = & \frac{-{\varOmega }^{2}EI[\cosh (\varOmega l)-\,\cos (\varOmega l)]}{1-\,\cos (\varOmega l)\cosh (\varOmega l)}\\ {V}_{l}^{^{\prime\prime} } & = & \frac{-{\varOmega }^{2}EI[\sinh (\varOmega l)\sin (\varOmega l)]}{1-\,\cos (\varOmega l)\cosh (\varOmega l)}\\ {M}_{0}^{^{\prime\prime} } & = & \frac{-\varOmega EI[\sin (\varOmega l)-\,\sinh (\varOmega l)]}{1-\,\cos (\varOmega l)\cosh (\varOmega l)}\\ {M}_{l}^{^{\prime\prime} } & = & \frac{-\varOmega EI[\cosh (\varOmega l)\,\sin (\varOmega l)-\,\cos (\varOmega l)\sinh (\varOmega l)]}{1-\,\cos (\varOmega l)\cosh (\varOmega l)}\end{array}$$

Corresponding to Fig. 7b, the expressions of shear forces and bending moments at node $i$ are obtained as follows:

12 $$\begin{array}{rcl}{V}^{-} & = & -\,{V}_{0}^{^{\prime} }{w}_{i}{e}^{-{\rm{i}}kl}+{V}_{l}^{^{\prime} }{w}_{i}+{V}_{0}^{^{\prime\prime} }{\phi }_{i}^{+}{e}^{-{\rm{i}}kl}+{V}_{l}^{^{\prime\prime} }{\phi }_{i}^{-}\\ {V}^{+} & = & {V}_{0}^{^{\prime} }{w}_{i}{e}^{{\rm{i}}kl}-{V}_{l}^{^{\prime} }{w}_{i}+{V}_{0}^{^{\prime\prime} }{\phi }_{i}^{-}{e}^{{\rm{i}}kl}+{V}_{l}^{^{\prime\prime} }{\phi }_{i}^{+}\\ {M}^{-} & = & {M}_{0}^{^{\prime} }{w}_{i}{e}^{-{\rm{i}}kl}+{M}_{l}^{^{\prime} }{w}_{i}-{M}_{0}^{^{\prime\prime} }{\phi }_{i}^{+}{e}^{-{\rm{i}}kl}+{M}_{l}^{^{\prime\prime} }{\phi }_{i}^{-}\\ {M}^{+} & = & {M}_{0}^{^{\prime} }{w}_{i}{e}^{{\rm{i}}kl}+{M}_{l}^{^{\prime} }{w}_{i}+{M}_{0}^{^{\prime\prime} }{\phi }_{i}^{-}{e}^{{\rm{i}}kl}-{M}_{l}^{^{\prime\prime} }{\phi }_{i}^{+}\end{array}$$

The kinematic compatibility condition for rotation is given by

13 $${\phi }_{i}^{+}={\phi }_{i}^{-}$$

and for equilibrium at node $i$ (Fig. 7e), the bending moments and forces equations are given as

$${M}^{+}={M}^{-}$$

14 $${V}^{+}={V}^{-}+({K}_{v}-m{\omega }^{2}){w}_{i}$$

from the above Eqs. (12), (13) and (14), the linear homogeneous equations are obtained in terms of ${w}_{i}$ and ${\phi }_{i}^{+}$ as follows,

15 $$[2{M}_{0}^{^{\prime} }\,\sinh ({\rm{i}}kl)]{w}_{i}+[2{M}_{0}^{^{\prime\prime} }\,\cosh ({\rm{i}}kl)-2{M}_{l}^{^{\prime\prime} }]{\phi }_{i}^{+}=0$$

16 $$[2{V}_{0}^{^{\prime} }\,\cosh ({\rm{i}}kl)-2{V}_{l}^{^{\prime} }-{K}_{v}+m{\omega }^{2}]{w}_{i}+[2{V}_{0}^{^{\prime\prime} }\,\sinh ({\rm{i}}kl)]{\phi }_{i}^{+}=0$$

where, ${K}_{v}$ and *m* are lateral stiffness of rack column and lumped mass of the rack, respectively. Equations (15) and (16) can be written as,

17 $$Hu=0$$

where, $$u={({w}_{i},{\phi }_{i}^{+})}^{T}$$ and

$$H=\left[\begin{array}{cc}2{M}_{0}^{{\rm{^{\prime} }}}\,\sinh ({\rm{i}}kl) & 2{M}_{0}^{{\rm{^{\prime} }}{\rm{^{\prime} }}}\,\cosh ({\rm{i}}kl)-2{M}_{l}^{{\rm{^{\prime} }}{\rm{^{\prime} }}}\\ 2{V}_{0}^{{\rm{^{\prime} }}}\,\cosh ({\rm{i}}kl)-2{V}_{l}^{{\rm{^{\prime} }}}-{K}_{v}+m{\omega }^{2} & 2{V}_{0}^{{\rm{^{\prime} }}{\rm{^{\prime} }}}\,\sinh ({\rm{i}}kl)\end{array}\right]$$

For a non- trivial solution of $u$, the determinant of $H$ must be zero i.e.,

18 $$\left|\begin{array}{cc}2{M}_{0}^{\prime}\,\sinh ({\rm{i}}kl) & 2{M}_{0}^{{\prime}{\prime}}\,\cosh ({\rm{i}}kl))-2{M}_{l}^{\prime{\prime}}\\ 2{V}_{0}^{{\prime}}\,\cosh ({\rm{i}}kl)-2{V}_{l}^{{\prime} }-{K}_{v}+m{\omega}^{2} & 2{V}_{0}^{{\prime}{\prime}}\,\sinh ({\rm{i}}kl))\end{array}\right| =0$$

Solving Eq. (18), the dispersion relation for periodic piping system is obtained as,

19 $$[2{M}_{0}^{{\rm{^{\prime} }}}{V}_{0}^{{\rm{^{\prime} }}{\rm{^{\prime} }}}{\sinh }^{2}({\rm{i}}kl)]-[2{V}_{0}^{{\rm{^{\prime} }}}\,\cosh ({\rm{i}}kl)-2{V}_{l}^{{\rm{^{\prime} }}}-{K}_{v}+m{\omega }^{2}][{M}_{0}^{{\rm{^{\prime} }}{\rm{^{\prime} }}}\,\cosh ({\rm{i}}kl)-{M}_{l}^{{\rm{^{\prime} }}{\rm{^{\prime} }}}]=0$$

### Numerical model

A finite element model of the periodically supported pipe $P$ is made using a two-dimensional Euler-Bernouli beam available in ANSYS APDL 19.0. To investigate the propagation of wave of frequency $f=\omega /2\pi$ in $P$, a harmonic rotation ${\phi }_{i/p}{e}^{{\rm{i}}2\pi ft}$ is applied at left end of $P$ and the steady state response ${\phi }_{o/p}(f)$ is measured at the right end. The vibration transmission behaviour is described by ${T}_{\phi }\,(dB)$, which is defined as

20 $${T}_{\phi }=20\,lo{g}_{10}\left|\frac{{\phi }_{o/p}(f)\,}{{\phi }_{i/p}(f)}\right|$$

The mesh size of finite element model in homogeneous solids is calculated by the Courant number^40^. In one dimensional wave propagation, the velocity in the numerical model (${c}_{FEM}$) should be same as that in the real structure ($c$). For a numerical model with mesh size $\varDelta x$ and wavelength $\,\lambda$, this condition is approximately satisfied^41^ (${c}_{FEM}/c=0.99$) when $\lambda /\varDelta x=16$.

$c=\sqrt{G/\rho }$, where $G$ and ρ are modulus of rigidity and density of the material, respectively. Based on the above formula, for a frequency of $200\,Hz$, mesh size is calculated to be 1 m. The transmission of vibration in $P$ is measured here using ${T}_{\phi }$ which depends on the number of unit cells. Since an infinite periodic structure cannot be considered in a numerical simulation, therefore, in order to validate the Floquet-Bloch theory presented in this paper, a finite pipe consisting of 40 unit cells is considered.

### Vibration control in periodic pipes

Let ${m}_{t,\alpha }$, ${k}_{t,\alpha }$ and ${c}_{t,\alpha }$ corresponds to the mass, stiffness and damping coefficient of the LLR. The optimal value ${k}_{t,\alpha }$ and ${c}_{t,\alpha }$ for a mass ratio $\alpha ={m}_{t,\alpha }/{(\rho Al)}_{P}$ can be obtained by minimizing the ${\Vert H\Vert }_{\infty }$ (peak value) of ${\phi }_{o/p}(f)$ of $P$. Closed form expressions exist for ${k}_{t,\alpha }$ and ${c}_{t,\alpha }$ when the main structure is undamped and is a single degree of freedom system^30,31^. Since the structure considered here is a continuous system with material damping $\xi$, a rigorous optimization study is essential to determine the optimal parameters. Let ${\Vert {H}_{Control}\Vert }_{\infty }$ and ${\Vert {H}_{Uncontrol}\Vert }_{\infty }$ refer to the peak value of ${\phi }_{o/p}(f)$ in the configuration with and without LLR, respectively. The optimal values for a particular $\alpha$ is obtained by performing optimization using genetic algorithm^42,43^ as follows,

$$\{{k}_{t,\alpha },{c}_{t,\alpha }\}=\text{arg}\,\min (Z)$$

subjected to,

21 $$\{LB\}\le \{{k}_{t,\alpha },{c}_{t,\alpha }\}\le \{UB\}$$

where

22 $$Z={\Vert {H}_{Control}\Vert }_{\infty }/{\Vert {H}_{Uncontrol}\Vert }_{\infty }$$

$\{LB\}$ and $\{UB\}$ respectively represents the lower and upper bound for ${k}_{t,\alpha }$ and ${c}_{t,\alpha }$. The values of these bounds are chosen such that the optimal ${k}_{t,\alpha }$ and ${c}_{t,\alpha }$ do not take unrealistic values and thus result in a faster optimization. A similar genetic algorithm-based optimization is used to determine the optimal spring-damper parameters (${k}_{{P}_{1,\alpha }}$ and ${c}_{{P}_{1,\alpha }}$) when ${P}_{1,\alpha }$ with $\alpha ={(\rho Al)}_{{P}_{1,\alpha }}/{(\rho Al)}_{P}$ is used instead of a LLR as shown in Fig. 2c.
